# Benevolent Sexism and the Traditional Sexual Script as Predictors of Sexual Dissatisfaction in Heterosexual Women from the U.S.

**DOI:** 10.1007/s10508-022-02318-3

**Published:** 2022-07-05

**Authors:** Sarah Bonell, Harrison Lee, Samuel Pearson, Emily Harris, Fiona Kate Barlow

**Affiliations:** 1 Melbourne School of Psychological Sciences, The University of Melbourne, Melbourne, 3010 Australia; 2 School of Psychology, The University of Queensland, Brisbane, Australia

**Keywords:** Benevolent sexism, Sexism, Traditional sexual script, Sexual satisfaction, Women’s sexual health

## Abstract

Women report lower sexual satisfaction than men. Given that sexual dissatisfaction adversely impacts health and well-being, it is imperative that we investigate why women are sexually dissatisfied. In the present study, we explored whether women’s benevolently sexist attitudes might predict their sexual dissatisfaction. In a sample of 308 (*M*_age_ = 38.09) heterosexual American women who had previously had sex with a man, we hypothesized that women’s benevolent sexism would be associated with an increased adoption of the traditional sexual script (i.e., an increased propensity for submissiveness and passivity during sex) and that this, in turn, would be associated with increased sexual dissatisfaction. We also hypothesized that the relationship between the adoption of the traditional sexual script and sexual dissatisfaction would be moderated by the degree to which participants enjoy submissiveness. Overall, we did not find support for our model: benevolent sexism did not predict sexual dissatisfaction. However, we did find that adopting the traditional sexual script was predictive of sexual dissatisfaction for women who do not enjoy submissiveness. These findings contribute to an emerging literature pertaining to women’s sexual health. Specifically, results suggest that benevolent sexism does not contribute to women’s experiences of sexual dissatisfaction. Instead, they suggest that sexual dissatisfaction in women may (in part) be driven by their engagement in sexual roles that do not align with their sexual preferences. Theoretical and clinical implications for these findings are discussed.

## Introduction

Sexual satisfaction is a core element of health and well-being (Mulhall et al., 2008; Stephenson & Meston, 2015). Being sexually satisfied is associated with greater life satisfaction and relationship satisfaction, as well as reduced risk of anxiety and depression (Carcedo et al., 2020; McNulty et al., 2016; Stephenson & Meston, 2015). Therefore, investigating factors that play a role in women’s sexual satisfaction is important, especially when considering that some literature suggests women are less sexually satisfied than men across different sexual contexts and relationship (Carpenter et al., 2009; Mark et al., 2015). In part, gender differences in sexual satisfaction may be attributable to physiological factors (e.g., women report greater difficulty orgasming relative to men, Frederick et al., 2017). Part of this disparity, however, is also likely to reflect social disparities (i.e. the different sexual pressures and expectations placed on men and women; Harris et al., 2016). In the present study, we explore the links between heterosexual women’s beliefs about gender and sex and sexual dissatisfaction.

### Gender Beliefs and Benevolent Sexism

Gender beliefs describe people’s expectations for how women and men should move through the world (Sanchez et al., 2005). A common way of examining the influence of gender beliefs on attitudes and behaviors in psychological research is by assessing the extent to which people endorse sexist worldviews (Glick & Fiske, 1996). Benevolent sexism describes a set of gender beliefs that are subjectively positive about women but ultimately undermine women’s power, competence, and autonomy (Glick et al., 2015; Harris et al., 2016). For example, the belief that men should look after women characterizes benevolent sexism; is positive in that it encourages a kind, benevolent disposition towards women, but it is patronizing in that it implicitly assumes that women need men to look after them. Unlike hostile sexism (i.e. overtly disliking women who violate or challenge traditional gender stereotypes), benevolently sexist attitudes are as prevalent in women as they are in men (Glick & Fiske, 1996; Glick et al., 2000; Harris et al., 2016). Research on the role of benevolent sexism in societal and relational settings underscores a number of negative consequences of benevolent sexism for women; for example, women who are benevolently sexist struggle to persevere in relationships when problems occur (Hammond & Overall, 2013). However, research on the role of benevolent sexism in women’s sexual experiences, and sexual satisfaction, is in its infancy.

We propose that the negative consequences of benevolent sexism for women may extend to their sexual experiences. A benevolently sexist worldview positions women as pure, moral, and refined; conversely, men are positioned as protectors and admirers, who should cherish and adore women and place them on a pedestal. One consequence of positioning women as sexually pure may be that women are expected to be sexually passive, in control of their sexual desire, and less motivated by sexual pleasure than men. Given that existing literature has established an association between sexual passivity and sexual dissatisfaction (Sanchez et al., 2011, 2012a, b), we theorize that women who hold a benevolently sexist worldview may be dissatisfied with their sex lives, since they may take a passive role during sex that prioritizes their partner’s pleasure over their own.

To our knowledge, there is only one study to date that has explicitly examined the association between benevolent sexism and sexual dissatisfaction for women. Among a sample of women of diverse sexualities (e.g., heterosexual, homosexual), Lentz and Zaikman (2021) found no direct link between benevolent sexism and sexual dissatisfaction. However, based on related existing literature, we contend that benevolent sexism might indirectly predict sexual dissatisfaction specifically for heterosexual women. For example, Harris et al. (2016) examined whether benevolent sexism was predictive of orgasm frequency for heterosexual women. While they established no direct link, benevolent sexism was indirectly associated with orgasm frequency. Specifically, benevolently sexist women perceived men as more sexually selfish; in turn, they were less willing to ask men for sexual pleasure and subsequently orgasmed less frequently. Given that orgasm frequency has been shown to positively predict sexual satisfaction (Haning et al., 2007), we propose that benevolent sexism may also indirectly predict sexual dissatisfaction. As such, the present study aims to examine the mechanism through which benevolent sexism might predict sexual dissatisfaction for heterosexual women. We propose that benevolent sexism may be associated with adherence to traditional (sexual) gender roles, and that this in turn might predict sexual dissatisfaction.

### The Traditional Sexual Script

In any social interaction, people must quickly and accurately assess how they are expected to behave, taking into account whether their behavior will be received positively or negatively by others. Given the sheer number of interactions individuals encounter within a lifetime, however, it is impossible for anyone to make these judgements on an entirely case-by-case basis. Instead, we come to rely on scripts–cognitive schemas or “playbooks” that provide guidelines for how to act in familiar and common situations (Abelson, 1981). Sexual activity is no exception (Simon & Gagnon, 1986, 2003).

The most common sexual script for heterosexual relationships in Western society is the traditional sexual script (O’Sullivan & Byers, 1992). In this script, men and women are expected to adhere to gender-specific behaviors during sexual activity. These behaviors are consistent with stereotypical gender roles (Masters et al., 2013). Men are assumed to have stronger sexual needs than women and are subsequently expected to initiate sexual contact (Byers, 1996; Littleton & Axsom, 2003; Masters et al., 2013). In contrast, women are considered the gatekeepers of sex—they are expected to succumb to men’s sexual urges by expressing initial reluctance, and subsequent submission, toward engaging in sex. Further, women are assumed to take on a passive and submissive role during intercourse (Byers, 1996; Wiederman, 2005).

### Benevolent Sexism and the Traditional Sexual Script

Given that the traditional sexual script encompasses stereotypically gendered sexual roles (O’Sullivan & Byers, 1992), we infer that women who endorse benevolent sexism will be more likely to adopt the traditional sexual script. No study to date has specifically explored the relationship between benevolent sexism and traditional sexual script adoption. However, existing literature has shown that benevolently sexist women are more likely to seek out sexually dominant partners (Altenburger et al., 2017; Harris et al., 2016). Furthermore, benevolent sexism in women is associated with valuing paternalistic chivalry—that is, the tendency for men to assume a dominant role in courtship and sexual practices (Viki et al., 2003). As such, we propose that benevolently sexist women will be more likely to adopt the traditional sexual script.

### The Traditional Sexual Script, Sexual Preferences, and Sexual Dissatisfaction

A woman’s willingness to ask for sexual pleasure directly influences the frequency with which she orgasms (Harris et al., 2016), suggesting that women who are not willing to ask for sexual pleasure are also less likely to be sexually satisfied (Haning et al., 2007). Given that a woman asking for pleasure would deviate from the traditional sexual script (Masters et al., 2013), women adopting the traditional sexual script may be less sexually satisfied. Further, research suggests that conforming to gender norms and sexual submissiveness—much like is expected in the traditional sexual script—reduces sexual autonomy, thereby reducing sexual satisfaction and arousal (Sanchez et al., 2005, 2006). While sexual submissiveness might be associated with sexual dissatisfaction, some women prefer to be submissive during sex. Sanchez et al. (2012a, b) found that among such women, sexually submissive behavior did not lead to reduced sexual satisfaction; submissive behavior only reduced satisfaction among women who did not have a preference for submission. Therefore, we predict that traditional sexual script adoption will predict sexual dissatisfaction, but only among women with a low degree of interest in sexual submission.

### Present Study

**Fig. 1:**
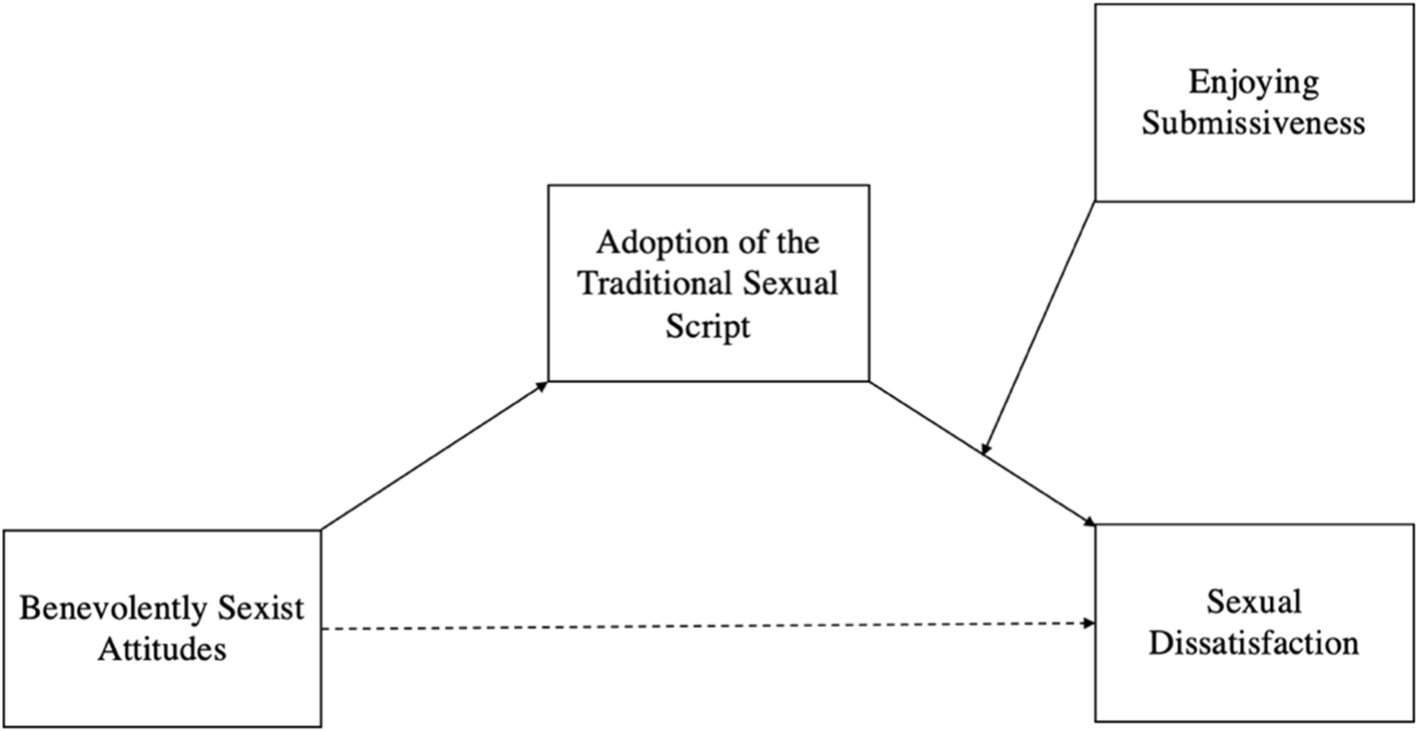
Proposed model in which benevolently sexist attitudes indirectly predict sexual dissatisfaction via the adoption of the traditional sexual script, and enjoying submissiveness moderates the relationship between adoption of the traditional sexual script and sexual dissatisfaction

In the present study, we investigate the relationship between benevolent sexism, the traditional sexual script, and sexual dissatisfaction in heterosexual women (Open Science Framework: https://tinyurl.com/5y443znp). We hypothesize that there will be an indirect relationship between benevolently sexist attitudes and sexual dissatisfaction, such that benevolent sexism will be associated with an increased adoption of the traditional sexual script, which in turn will be associated with increased sexual dissatisfaction. In line with Sanchez et al. (2012a, b), we also hypothesize that the relationship between traditional sexual script adoption and sexual dissatisfaction will be moderated by sexual preferences, such that adopting the traditional sexual script will only predict sexual dissatisfaction among women who do not enjoy being submissive. Finally, we hypothesize that our overall moderated mediation model (see Fig. 1) will account for a significant proportion of variance in heterosexual women’s sexual dissatisfaction.

## Method

Our study was pre-registered, and all materials and data are available for public access on the Open Science Framework (https://tinyurl.com/2bah9x5r). Ethics approval for the study was obtained from The University of Queensland prior to study commencement (Ethics ID: 2,020,000,032).

### Participants

After excluding 41 participants for failing one or both attention checks included in the study, there were 308 participants (*M*_age_ = 38.09 years, SD = 11.18). They were heterosexual American women who had had sex with a man. Participants were recruited via Amazon’s Mechanical Turk (MTurk). The number of participants recruited for this study was predominantly determined by funding available to researchers at the time of data collection. Participants were compensated with US$1.33 to complete the 10-min survey (see Buhrmester et al., 2011, for a review regarding the effect of compensation on data quality).

### Measures

#### The Ambivalent Sexism Inventory–Benevolent Sexism Subscale (ASI-BS)

The ASI-BS is a validated 11-item questionnaire that assesses benevolently sexist attitudes (Glick & Fiske, 1996). Participants rated the extent to which they agreed with each statement describing benevolently sexist attitudes (e.g., “In a disaster, women ought not necessarily to be rescued before men”) on a 6-point Likert scale (1 = *disagree strongly,* 6 = *agree strongly*). Three items were negatively worded and subsequently reverse scored. Missing data were handled using pairwise deletion. Responses were averaged to obtain a final score out of 6, with higher scores indicating more benevolently sexist attitudes. Internal consistency was excellent (*α* = 0.92).

### Adoption of the Traditional Sexual Script

As a proxy for the adoption of the traditional sexual script, participants completed a previously validated 4-item questionnaire to assess the degree to which they engage in sexually submissive behaviors (see Sanchez et al., 2006, 2012a, b). Participants rated on a 7-point Likert scale (where 1 = *strongly disagree,* 7 = *strongly agree*) the extent to which they agreed with a series of four statements describing engagement with sexual submissiveness (e.g., “I tend to take on the more passive role during sexual activity”). One item was negatively worded and subsequently reverse scored. Missing data was handled using pairwise deletion. Responses were averaged to obtain a final score out of 7 for each participant, with higher scores indicating that a participant engaged in more sexually submissive behavior. Internal consistency was good (*α* = 0.88).

### Global Measure of Sexual Satisfaction–Revised (GMSEX)

The GMSEX is a validated 5-item questionnaire that assesses sexual satisfaction (Glick & Fiske, 1996). Participants were asked to rate their sexual relationship(s) with men on five 7-point bipolar scales: good–bad, pleasant–unpleasant, positive–negative, satisfying–unsatisfying, and valuable–worthless. Missing data were handled using pairwise deletion. Responses were averaged to obtain a final score out of 7, with higher scores indicating greater sexually dissatisfaction. Internal consistency was excellent (*α* = 0.95).

#### Sexual Preference for Submission

To assess participant preference for submissiveness, we used a previously validated 3-item questionnaire designed to assess the degree to which individuals enjoy having a sexually dominant partner (see Sanchez et al., 2012a, b). Participants rated on a 7-point Likert scale (where 1 = *strongly disagree,* 7 = *strongly agree*) the extent to which they agreed with the following statements: “I find it arousing when my partner is the aggressive one in bed,” “I think it is sexy when my partner takes control in bed,” and “I think it is very exciting when my partner leads our sexual experiences.” Responses were averaged to obtain a final score out of 7, with higher scores indicating a stronger sexual preference for submission. Internal consistency was excellent (*α* = 0.92).

### Procedure

We first conducted a screening questionnaire such that only participants who identified as female and heterosexual, were 18 or older, and reported having had sex with a man were invited to take part in the main survey (compensation USD$0.05). Eligible participants were informed that the study involved answering sensitive questions (e.g., questions pertaining to their sexual beliefs and satisfaction), and were asked if they wished to continue (for further compensation of USD$1.28). If they chose to proceed, participants were provided with a link to an online survey where they were presented with further details of the study and were asked to provide consent. We included an attention check item asking consenting participants to select the purpose of the study from a list of four options. Participants were then asked to report their age in years. Next, in a randomized order, participants completed the ASI-BS, adoption of the traditional sexual script measure, GMSEX, and Sexual Preference for Submission measure. Within the survey, participants were presented with a second attention check item instructing them to select the response, “strongly disagree.” Finally, participants were debriefed regarding the purpose of the study.

### Analyses

A total of 15 outliers were identified in our dataset across two variables—sexual dissatisfaction and enjoying submissiveness. Outlier calculations are available on the Open Science Framework (https://tinyurl.com/2bah9x5r). Analyses were conducted including and excluding outliers, and the results were substantively unchanged. Outliers were therefore retained in the main analysis. The hypothesized moderated mediation model (see Fig. 1) was tested in R Studio Version 1.4.1717 using the “PROCESS” package (Hayes, 2013).

## Results

### Descriptive Statistics

As shown in Table 1, there was no significant association between benevolently sexist attitudes and the adoption of the traditional sexual script, nor was there a significant relationship between benevolently sexist attitudes and sexual dissatisfaction. However, there was a significant positive relationship between the adoption of the traditional sexual script and sexual dissatisfaction; that is, participants who adopted the traditional sexual script were more sexually dissatisfied.

**Table 1:** Descriptive statistics matrix

Variable	*M*	SD	BS	TSS	SEXD
Benevolently sexist attitudes^BS^	3.15	1.17			
Adoption of the traditional sexual script^TSS^	4.73	1.32	.04 [− .07, .15]		
Sexual dissatisfaction^SEXD^	2.50	1.23	− .02 [− .13, .10]	.14^*^ [.03, .25]	
Enjoying submissiveness^ES^	5.61	1.20	.16^*^ [.05, .27]	.24^*^ [.14, .35]	− .14^*^ [− .25, − .03]

^*^p < .05, Range^BS^ = 1–6; Range^TSS^ = 1–7; Range^SEXD^ = 1.2–7; Range^ES^ = 1–7

### Testing the Hypothesized Model

**Fig. 2:**
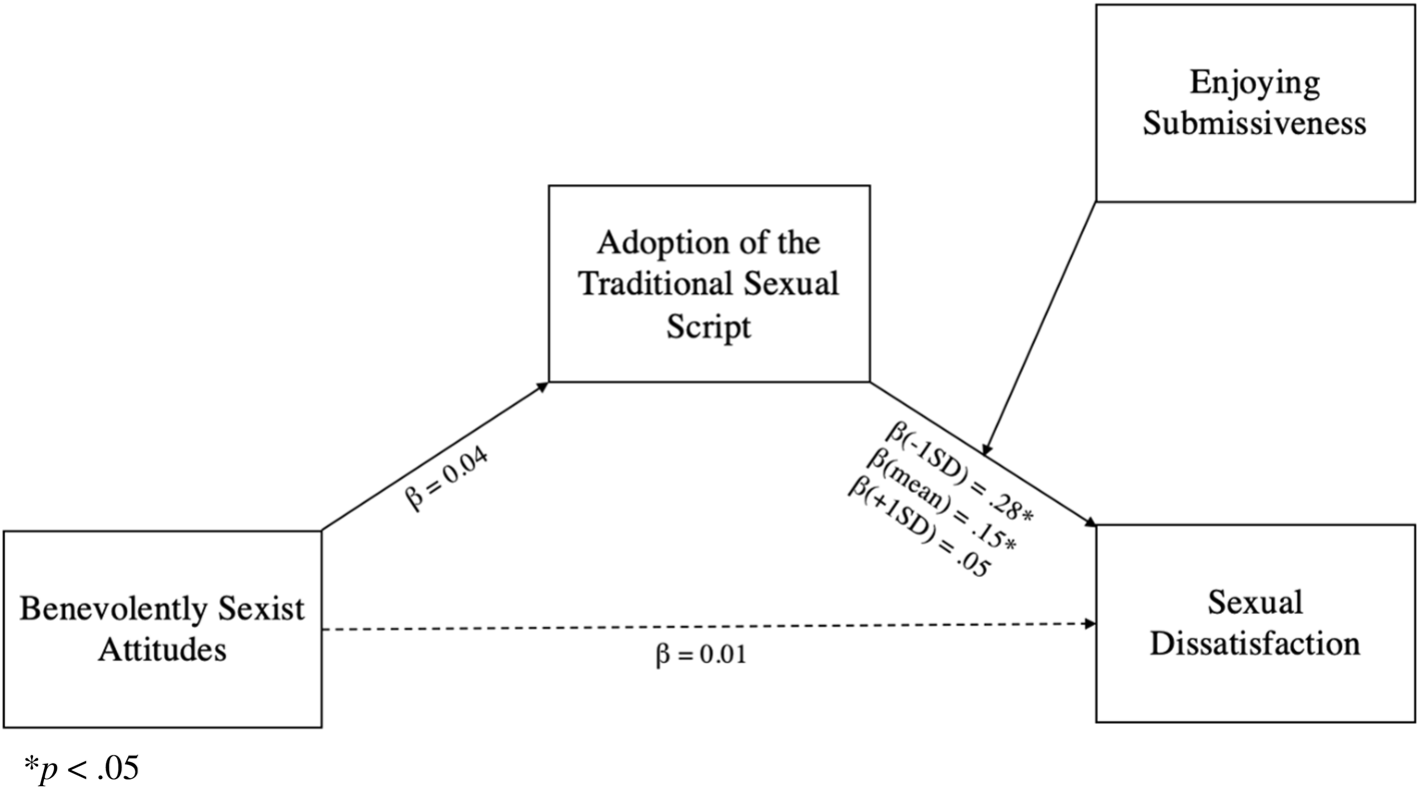
Conditional indirect effect of benevolent sexism on sexual dissatisfaction via adoption of the traditional sexual script, at Low (− 1SD), Average (Mean), and High (+ 1SD) levels of enjoying submissiveness. ^*^p < .05

Overall, our moderated mediation model did not account for a significant proportion of heterosexual women’s sexual dissatisfaction (see Fig. 2; index of moderated mediation = − 0.005; 95%CI = − 0.02, 0.01).

There was no indirect relationship between benevolently sexist attitudes and sexual dissatisfaction via the adoption of the traditional sexual script. However, as shown in Fig. 2, there was a significant relationship between traditional sexual script adoption and sexual dissatisfaction (*β* = 0.19, SE = 0.06, *t* = 3.24, *p* = .003), which was moderated by sexual preferences (*β* = − 0.12, SE = 0.05, *t* = − 2.27, *p* = .024). Specifically, the adoption of the traditional sexual script predicted sexual dissatisfaction only for participants who did not enjoy submissiveness (i.e. − 1SD, *p* < .001, or mean score, *p* = .015); for participants who enjoyed submissiveness (i.e. + 1SD), adopting the traditional sexual script did not predict sexual dissatisfaction (*p* = .562). Overall, while our model was generally not supported, we found that adopting the traditional sexual script was associated with sexual dissatisfaction for women who do not enjoy submissiveness.

## Discussion

In the present study, we investigated the relationships between benevolent sexism, the traditional sexual script, and sexual dissatisfaction in heterosexual women. Contrary to our predictions, our moderated mediation model did not account for a significant proportion of heterosexual women’s sexual dissatisfaction. Furthermore, there was no indirect relationship between benevolently sexist attitudes and sexual dissatisfaction via the adoption of the traditional sexual script. These findings contribute to a small number of studies examining the links between benevolent sexism and measures of sexual satisfaction and enjoyment, which collectively suggest that benevolent sexism is not directly associated with sexual satisfaction (Harris et al., 2016; Lentz & Zaikman, 2021).

We theorized that benevolent sexism likely has some negative consequences for women’s sexual satisfaction; however, it is possible that women’s conceptualizations of sexual satisfaction vary in ways that lead to parity in sexual satisfaction among women high and low in benevolent sexism. It may be that women higher in benevolent sexism derive sexual satisfaction from factors not necessarily associated with physical pleasure. A thematic analysis of people’s definitions of sexually satisfaction found that sexual satisfaction can comprise two subcomponents: individualistic satisfaction (i.e., that associated with pleasure, arousal, and orgasm) and relational satisfaction (i.e., that associated with romance, expression of feeling, and creativity; Pascoal et al., 2014). Since a benevolently sexist worldview suggests that women should desire to feel adored and cherished by men (Glick & Fiske, 1996), women higher in benevolent sexism may derive greater satisfaction from the intimacy associated with having sex and/or the commendation they receive from partners for engaging in sex (i.e., relational factors), rather than from sexual pleasure itself (i.e., individualistic factors; Pascoal et al., 2014). As such, benevolently sexist attitudes may not predict sexual dissatisfaction but rather shape and color women’s definitions of what it means to be sexually satisfied. Future studies may explore the role of benevolent sexism in shaping definitions of sexual satisfaction by using primes to experimentally manipulate benevolently sexist attitudes among women and assessing whether sexual satisfaction is conceptualized differently across groups (i.e., whether valuations of individualistic and relational factors change as a function of benevolently sexist attitudes; Pascoal et al., 2014).

Additionally, we may not have found an association between benevolent sexism and sexual dissatisfaction because certain facets of benevolent sexism might increase sexual satisfaction and suppress possible mechanisms that may be associated with increased dissatisfaction. As noted by Harris et al. (2016), benevolently sexist women may be drawn to stereotypically “masculine” sexual partners, given their preferences for traditional gender roles (Backus & Mahalik, 2011). As research suggests women are more likely to orgasm when having sex with masculine partners (Puts et al., 2012), negative effects of benevolent sexism for orgasm frequency (e.g., the belief that men are selfish in bed) may be cancelled out (Harris et al., 2016). Thus, the relationship between benevolent sexism and sexual dissatisfaction may be more nuanced than we had initially predicted; while benevolent sexism might act to repress female sexuality in some ways, in others it may enhance female sexual satisfaction. Further research is necessary to examine the potential positive and negative implications of benevolent sexism for women’s sexual satisfaction.

While, overall, our model was not supported, we found support for our second hypothesis. Past literature has suggested that when women conform to gender norms—for example, by being sexually submissive—they experience reduced sexual satisfaction and arousal because they experience less sexual autonomy (Sanchez et al., 2005, 2006). However, in line with Sanchez et al. (2012a, b), the present study found that adopting the traditional sexual script only predicted sexual dissatisfaction among women who reported they did not enjoy being submissive. As such, our findings suggest that the traditional sexual script in and of itself is not inherently predictive of female sexual dissatisfaction. The implications of these findings are twofold.

Firstly, our results challenge the theory that sexually submissive women are intrinsically less sexually autonomous (Sanchez et al., 2005, 2006). Instead, we contend that autonomy is oppressed in situations where women are denied their sexual preferences. As such, we recommend future research examine whether enjoying submissiveness moderates the relationship between female submissiveness and a loss of sexual autonomy. Secondly, our results have clinical implications. Namely, our findings emphasize the importance that sex therapists (and similar) need continue to place on encouraging women to explore and identify their sexual likes and dislikes. More specifically, the present study establishes that clinicians should not necessarily focus on discouraging women from engaging in gender-stereotypic sexual behaviors, but rather on encouraging them to reflect upon these norms to establish whether the traditional sexual script aligns with their own sexual preferences.

Our study was not without limitations. Firstly, we relied on a self-report cross-sectional design. As such, we were unable to establish causality in the present study. Further, we were unable to ask our participants how their gender beliefs may or may not relate to their sexual experiences. However, given that this project was graduate student-led, we did not have the resources available to conduct such substantiative follow-up studies. It is our hope that findings from the present study pave the way for larger future studies. As well as this, in the present study, we failed to control for how frequently participants had sex and/or their number of sexual partners. Further, we failed to control for benevolently sexist attitudes or sexual preferences among participants’ sexual partners as potentially confounding variables. Finally, our study was unable to represent the experiences of sexual minority women who engage in sexual activity with men. These women are vastly underrepresented in sexual health research, and it is still widely unknown whether and how they engage with the traditional sexual script. As such, future research ought to examine sexual minority women’s experiences of sexual dissatisfaction.

The present study highlights key avenues through which future research might gain a better understanding of how women come to be sexually dissatisfied. Namely, future research ought to determine (1) why some women fulfil sexual roles that do not align with their preferences and (2) how to encourage these women to pursue alternate sexual roles. Furthermore, research may examine how benevolent sexism influences women’s sexual experiences—in particular, how benevolent sexism might (1) encourage women to reconceptualize sexual satisfaction or (2) positively and negatively affect sexual satisfaction overall. In sum, this study contributes to ongoing scholarship aimed at empowering women to engage in fulfilling, engaging, and satisfying sexual activity.
